# Dielectrophoretic Profiling of *Candidozyma auris*: the Effect of Glucose on Cellular Polarizability

**DOI:** 10.1021/acsmeasuresciau.5c00084

**Published:** 2025-09-26

**Authors:** Negar Farhang-Doost, Camila S. Cué Royo, Tagbo H. R. Niepa, Soumya K. Srivastava

**Affiliations:** † Department of Chemical and Biomedical Engineering, 195919 West Virginia University, Morgantown, West Virginia 26506, United States; ‡ Department of Chemical Engineering, 6612 Carnegie Mellon University, Pittsburgh, Pennsylvania 15213, United States; § Department of Biomedical Engineering, Carnegie Mellon University, Pittsburgh, Pennsylvania 15213, United States

**Keywords:** *Candidozyma auris*, dielectrophoresis, dielectric spectra, single-shell model, glucose metabolism

## Abstract

*Candidozyma auris* is an emerging multidrug-resistant fungal pathogen that poses significant challenges to healthcare systems worldwide. Its ability to persist on surfaces and resist common disinfectants contributes to rapid nosocomial transmission, making early and acute detection crucial for infection control. Conventional culture-based identification methods are time-consuming and lack sensitivity, while molecular techniques are expensive and require specialized equipment and trained personnel. This study explores the use of dielectrophoresis (DEP) for the rapid detection of *C. auris* by quantifying its dielectric properties using the dielectric single-shell model. Furthermore, since glucose plays a fundamental role in yeast metabolism, including in *C. auris*, we investigate how glucose metabolism affects its dielectric behavior. Changes in ionic concentrations and enzyme activity induced by glucose metabolism can alter the electrical properties of *C. auris* cells, making them more responsive to external electric fields. By characterizing these dielectric shifts under glucose-rich and glucose-limited conditions, we aim to develop a DEP-based diagnostic platform for the rapid and label-free detection of *C. auris*. This approach could provide an effective alternative to current diagnostic methods, enhancing screening efforts and improving infection control in healthcare settings.

## Graphical abstract

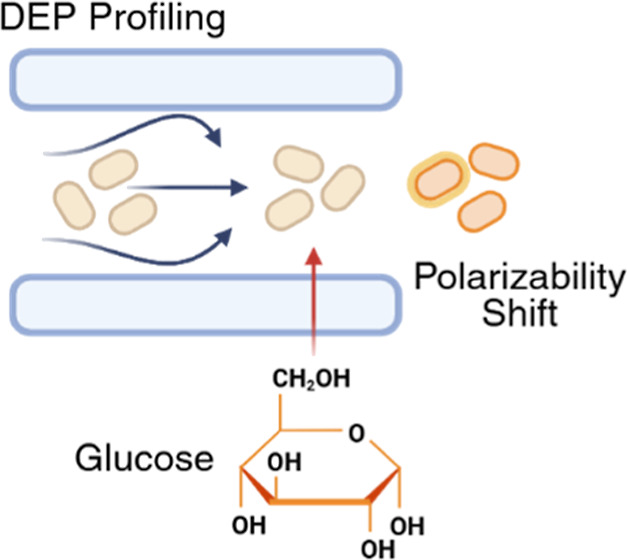

## 1 Introduction

*Candidozyma auris* is a type of yeast that can cause severe illness and was first reported in Japan in 2009.^1^ Previously referred to as *Candida auris*, the pathogen has been reclassified to highlight phylogenetic distance from *Candida* species. The emergence of *C. auris* has become a serious global health issue owing to its clonal intercontinental spread.^1,2^ The ability of *C. auris* to survive on surfaces and resist common disinfectants contributes to its spread within healthcare facilities, posing a challenge to infection control efforts.^3^ In 2022, there were 2377 clinical cases and 5754 screening cases in the United States. *C. auris* poses significant challenges in both diagnosis and treatment by causing severe, multidrug-resistant, and difficult-to-cure infections. Some strains of *C. auris* are resistant to all four available classes of antifungals: azoles (via ERG11 and TAC1B mutations), polyenes (via ERG11 mutation),^4^ echinocandins^5,6^ (through FKS1 hotspot alterations), and allylamine/thiocarbamates.^7^

Current culture-based methods for isolating *C. auris* are slow and lack sensitivity. Although conventional culture methods are economical and easier to implement without specialized equipment, they remain hindered by slow yeast growth and the need for MALDI-ToF databases for accurate identification.^8^

Additionally, distinguishing *C. auris* from other *Candida* species on standard agar plates is challenging,^11^ necessitating the examination of every suspicious colony and making it time-intensive. In contrast, molecular methods offer greater sensitivity but require sample preparation, specialized equipment, and fluorescent tags, which makes it expensive. Thus, there is an urgent demand for accessible and rapid identification methods to enhance the screening of patients. To address the limitations associated with current identification methods, this study employs an electrokinetic technique, dielectrophoresis (DEP), to detect *C. auris* by characterizing its behavior under nonuniform electric fields, yielding quantifiable dielectric properties using the dielectric single-shell model. DEP presents a promising approach for developing a cost-effective, label-free, and sensitive point-of-care diagnostic tool.^12^ DEP is cost-effective, primarily because it is label-free, eliminating the need for expensive reagents like fluorescent antibodies and primers. While a new flow cytometer or qPCR machine can be a major investment with significant ongoing costs, DEP offers a more accessible and affordable alternative, especially for laboratories with limited budgets. The time efficiency of dielectrophoresis (DEP) is a key advantage over multistep methods such as MALDI-ToF.^9,10^ Another advantage is the label-free nature of DEP, as it avoids the need for costly and time-consuming staining and tagging procedures, which can also affect cell viability.^10,11^ The third benefit of DEP is its low cost, which makes it suitable for use outside of a conventional laboratory setting.^11^ Table 1 demonstrates the cost comparison of DEP, flow cytometry, and qPCR.

**Table 1:** Cost Comparison of DEP, Flow Cytometry, and qPCR

Method	Initial Equipment Cost	Recurring Reagent Cost	Pros & Cons
Dielectrophoresis (DEP)^13^	variable (can be low with simple designs)	minimal (label-free)	reduced operational cost, fast, label-free, and maintains cell viability.
Flow Cytometry	very high ($10,000s–$100,000s)	high (fluorescent antibodies)	highly specific, high throughput, but expensive, and can damage cells.
Real-time PCR (qPCR)	high ($15,000–$150,000)	moderate to high (primers, master mixes)	highly sensitive, quantitative, but requires expensive reagents and specialized equipment.

Exploring the dielectric properties of *C. auris* enables the design of a label-free point-of-care DEP platform deployable under low-resource settings for the rapid detection of *C. auris*.

Glucose is a key carbon source for yeast cells, providing the primary fuel for energy production and supporting growth and reproduction across many biological systems.^14–17^ As a preferred sugar, glucose drives major metabolic pathways that allow rapid cell growth and carefully control cellular activities.^15,17^ In yeast, especially in the pathogenic species, *C. auris*, glucose is vital for activating critical energy processes, like the TCA (tricarboxylic acid) cycle and mitochondrial respiration, which adjust energy production between fermentation and respiration based on how much glucose is available.^16,17^ For *C. auris*, glucose supports survival and growth, helping the organism adapt to different environments, such as those inside a host.^18^ When yeast cells, like *C. auris*, metabolize glucose, they experience ionic and enzymatic changes that can modify their dielectric properties.^19,20^ The higher internal ion concentrations during glucose metabolism and changes in enzyme activity, particularly in the electron transport chain and TCA cycle, increase the cells’ responsiveness to electric fields.^21^ In this work, glucose acts primarily as a metabolic driver to accentuate cellular activity; however, our diagnostic premise is not based on the glucose response alone. Rather, we analyze the resulting electrokinetic behavior of *C. auris* across a broad frequency sweep, quantifying conductivity, permittivity, and particularly crossover frequency. These parameters together generate a dielectric fingerprint that is highly sensitive to cell size, morphology, membrane composition, and internal structure. These frequency-dependent dielectric profiles enable discrimination of *C. auris* from other pathogens even under shared metabolic conditions.

## 2 Theory

Dielectrophoresis (DEP) is a phenomenon that describes the field-induced force exerted on a polarizable particle within a nonuniform electric field.^20^ Dielectrophoresis (DEP) has been applied to characterize, enrich, and sort targeted cells ranging from DNA to mammalian cells.^11,22–30^ During DEP, cells are subjected to an oscillating electric field. As a result of the transient electric dipoles that form, the cells will either be attracted (positive dielectrophoresis or pDEP) to or repelled from regions with high electric field gradients (negative dielectrophoresis or nDEP), depending on the frequency of the applied field (Figure 1).^13^ During the transition between nDEP and pDEP, cells encounter a point where the DEP force becomes zero, known as the crossover frequency.^22^

**Figure 1:**
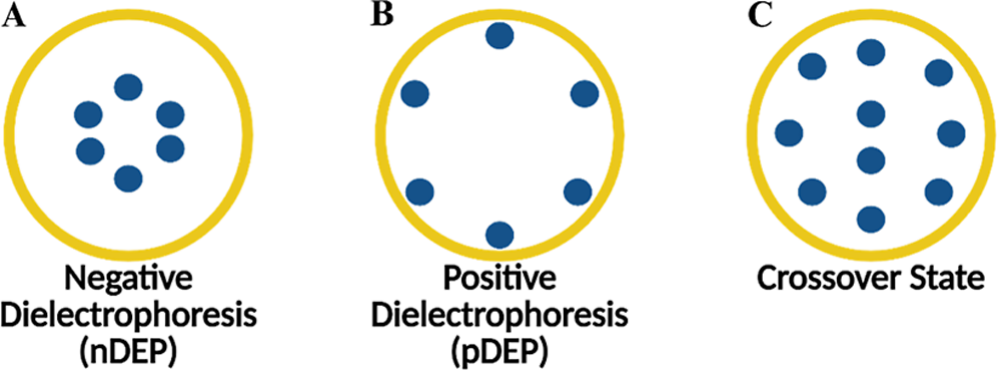
Schematic representation of cell behavior under dielectrophoresis. Cell (blue spheres) behavior under a nonuniform electric field in the microwell (yellow circles) of the 3DEP dielectrophoretic analyzer (DepTech, Uckfield, U.K.). (a) negative dielectrophoresis (nDEP) where the cells move toward the center (low electric field region) in a 3DEP microwell with gold-plated copper electrode along with the electric field lines originating at the walls of the microwell, (b) positive dielectrophoresis (pDEP) where the cells are attracted to the walls (high electric field region), and (c) crossover state where DEP force is zero. Figure created with Biorender.com.

DEP enables the assessment of the passive electrophysiological properties, such as conductance and capacitance, of a cell’s membrane and cytoplasm.^30^ The DEP force for a homogeneous sphere can be calculated using eq 1

1 $$F_{DEP}=2\pi r^{3}\varepsilon_{m}Re\left[K\left(\omega \right)\right]\nabla E^{2}$$

where *r* is the particle radius, ε_m_ is the permittivity of the suspending medium, ∇*E* is the gradient of the electric field, and *Re*[*K*(ω)] is the real part of the Clausius–Mossotti (CM) factor. The real part of the Clausius–Mossotti factor is frequency-dependent, which determines the magnitude and direction of the force acting on a cell under a nonuniform electric field. It depends on the dielectric properties of both the particle and the surrounding medium. The real part of the CM factor indicates whether the particle will experience pDEP or nDEP, and it becomes zero at the crossover frequency. CM factor is given by

2 $$Re\left[K\left(\omega \right)\right]=\frac{\varepsilon_{p}^{*}-\varepsilon_{m}^{*}}{\varepsilon_{p}^{*}+2\varepsilon_{m}^{*}}$$

where ε_p_* and ε_m_* are the complex permittivity of the particle and medium, respectively, that is given by

3 $$\varepsilon^{*}=\varepsilon -j\frac{\sigma }{\omega }$$

where ε is the permittivity, σ is the suspending medium conductivity, ω is the angular frequency of the applied field, and *j* is the complex number, $\sqrt{-1}$ . For a more complex structure like a cell, we can modify the parameter, ε_p_*, in eq 2 to a more intricate form that includes the properties of both the membrane and cytoplasm. This formulation is commonly known as the single-shell model and is expressed as follows^28,31–33^

4 $$\varepsilon_{cell}^{*}=\varepsilon_{mem}^{*}\frac{{\left(\frac{r}{r-t}\right)}^{3}+2\frac{\varepsilon_{cyto}^{*}-\varepsilon_{mem}^{*}}{\varepsilon_{cyto}^{*}+2\varepsilon_{mem}^{*}}}{{\left(\frac{r}{r-t}\right)}^{3}-\frac{\varepsilon_{cyto}^{*}-\varepsilon_{mem}^{*}}{\varepsilon_{cyto}^{*}+2\varepsilon_{mem}^{*}}}$$

where ε_mem_*, ε_cyto_*, and *t* are the complex permittivity of the membrane, complex permittivity of cytoplasm, and membrane thickness, respectively. The exploration of the specific dielectric properties of cells has been used to define cell health, physiological state, differentiation, function, and intracellular interactions.^22,27,28,30,34,35^

The single-shell model in dielectrophoresis is a widely used framework for describing the electrical properties of biological cells. It simplifies the cell as a homogeneous spherical particle, where the interior (cytoplasm) has its electrical characteristics and is surrounded by a shell representing the cell membrane. This model allows for the calculation of how cells interact with external electric fields, where cells experience forces based on the differences in electrical properties between their membrane, cytoplasm, and the surrounding medium.^36^ Figure 2 represents the single-shell model where *R* is the outer radius, *d* is the shell thickness, and *R* – *d* is the inner radius. In this study, we treated the cell wall and cell membrane of *C. auris* as a single shell due to the presence of both structures and to avoid complexity in analysis.

**Figure 2:**
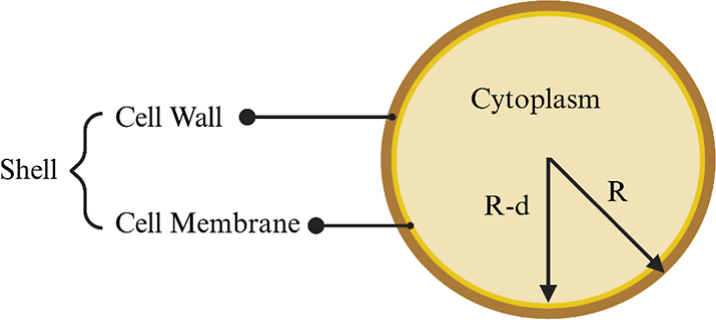
Single-shell model for a cell with radius “*R*” and shell thickness “*d*” that comprises both the membrane and cell wall. Each component, i.e., the shell and the cytoplasm, has its unique electrical signatures such as permittivity and conductivity. Figure created with Biorender.com.

## 3 Materials and Methods

### 3.1 Overnight Cell Culture

The wild-type *C. auris* AR 1100 (CA1100) cells, provided by the Centers for Disease Control and Prevention (CDC, USA), were cultured overnight for 18 h in a sterile Erlenmeyer flask containing 25 mL ultrafiltered tryptone–yeast extract medium containing UFTYE (ultrafiltered tryptone-yeast extract), 2.5% Bacto-Tryptone, 1.5% Bacto-Yeast, supplemented with 1% (wt/vol) glucose. The cultures were incubated at 37 °C with orbital shaking. Following the overnight incubation period, 10 mL of *C. auris* cell solution was collected and centrifuged at 6000 rpm for 10 min to separate cells from the media. Then, the cells were washed twice with 30 mL of 145 mM NaCl solution, followed by centrifugation at 6000 rpm for 10 min to remove cell debris and residual media.

To maintain consistent and reproducible results in all experiments, the cells in their stationary phase were suspended in the DEP suspending media (protocol in Section 3.4) to achieve an optical density at 600 nm (OD_600_) within the range of 0.6–0.8. The optical density was measured using the Evolution 300 (Thermo Scientific, USA), Cytation 5 multiwell plate reader (Agilent Technologies), and Evolution TM One UV–vis Spectrophotometer (Thermo Fisher, USA).

### 3.2 Kinetic Growth Study

This study evaluated the effects of glucose on the growth dynamics of *C. auris* strain CA1100. Growth curves were generated to determine how the presence or absence of glucose influences lag time, maximum growth rate (μ_max_), and growth potential (area under the curve, AUC).

A Falcon sterile flat-bottom 96-well plate (Corning Incorporated, NY) was prepared by adding 200 μL of UFTYE medium into each well of columns one and two and 200 μL of UFTYE with 1% (w/v) glucose to columns three and four. In columns two and four, 5 μL of a *C. auris* CA1100 overnight culture was dispensed into all wells, while columns one and three served as blanks for their respective conditions. The plate was then incubated at 37 °C for 48 h. A growth kinetic analysis was run using the Agilent Cytation 5 Cell Imaging Multimode Reader with the Gen5 Software (Agilent, USA). Optical density measurements at 600 nm (OD_600_) were recorded every 10 min to monitor growth kinetics. Following the experiment, the collected OD readings were exported to Excel and analyzed using the RStudio software to calculate lag time, μ_max_, and growth potential for both conditions.

### 3.3 Electron Microscopy

Two experimental conditions were studied, where CA1100 samples were exposed to a glucose-limited and a glucose-nonlimited solution. The glucose-supplemented sample was generated by adding 1% (wt/vol) glucose to the diluted *C. auris* cell solution. Glucose exposure was carried out at different time intervals of 20, 40, and 60 min.

#### 3.3.1 Scanning Electron Microscopy

Cell morphological differences were investigated by comparing scanning electron microscopy (SEM) images of glucose-limited and glucose-nonlimited *C. auris* cell samples.

An overnight *C. auris* strain CA1100 cell culture was prepared the day before exposing the cells to glucose and fixing them for SEM imaging. After 18 h, a 10 mL aliquot of the culture was centrifuged to separate the cells from the UFTYE medium containing 1% (w/v) glucose. The cells were washed with 145 mM NaCl and diluted to an OD_600_ of 0.65–0.75 in 145 mM NaCl solution. The diluted cell suspension was divided into two conical tubes. One tube was supplemented with 1% (w/v) glucose, while the other was maintained under glucose-limited conditions. The cells were incubated for 1 h at 37 °C, with samples taken at 20 min intervals.

After glucose exposure, the cells were prepared for SEM imaging. A 10 μL aliquot of the cell suspension was deposited onto titanium coupons, allowing the cells to adhere for 20 min. The adhered cells were then fixed in 2.5% glutaraldehyde for 2 h. Following fixation, the samples were washed three times with 145 mM NaCl (15 min per wash) to remove residual glutaraldehyde. The cells were then fixed again in 1% osmium tetroxide (OsO_4_) for 1 h, followed by three additional washes with 145 mM NaCl (15 min each). Dehydration was carried out through a graded ethanol series (30%, 50%, 70%, 90%, and 100%), with each concentration applied for 15 min, and the final step (100% ethanol) repeated twice. The samples were air-dried overnight in hexamethyldisilazane (HMDS). The next day, the dried samples were mounted onto specimen stubs for SEM analysis. The cell morphology was examined using a Tescan Mira 3 SEM at 10,000×, 20,000×, and 40,000× magnifications.

#### 3.3.2 Transmission Electron Microscopy

Cellular ultrastructure was analyzed by comparing transmission electron microscopy (TEM) images of *C. auris* cell samples subjected to glucose-limited and glucose-nonlimited conditions.

An overnight *C. auris* strain CA1100 culture was prepared and incubated for 18 h. A 10 mL aliquot of the culture was collected and centrifuged to remove the growth medium. The resulting pellet was resuspended in 2.5% glutaraldehyde prepared in 0.1 M phosphate-buffered saline (PBS, pH 7.4) and immediately centrifuged to form a pellet. The cells were fixed in the glutaraldehyde solution for at least 60 min.

Following fixation, the cells were washed once with 0.1 M PBS for 15 min and then washed three times with double-distilled (dd) water (ddH_2_O, 15 min per wash). Postfixation was performed by incubating the samples in 4% potassium permanganate (KMnO_4_) for 2 h, followed by three additional washes with ddH_2_O (15 min each). The specimens were further fixed and stained with 2% uranyl acetate for 12 h and rewashed three times with ddH_2_O (15 min each). Samples were then dehydrated through a graded ethanol series (30%, 50%, 70%, 90%, and 100%), with each concentration applied for 15 min, where the final 100% ethanol step was repeated three times. Additionally, specimens were further dehydrated using two 10 min changes of propylene oxide. Infiltration was performed with a 1:1 mixture of propylene oxide and Epon resin for 1–3 h, followed by three subsequent changes of pure Epon, each for 1 h. Lastly, the samples were embedded in pure Epon resin and cured overnight at 60 °C. The cured specimens were sectioned and mounted onto copper grids for TEM analysis. Cellular ultrastructure was observed using the JEOL JEM-1400Flash TEM at 30,000× and 40,000× magnifications to investigate differences between the samples under the two experimental conditions.

### 3.4 DEP Experiment Preparation

DI water was used as the DEP medium, and phosphate-buffered saline (PBS) was added to adjust the suspending medium conductivity to 80 μS/cm using a conductivity meter (InLab 731-ISM, Mettler Toledo, USA). For the glucose-supplemented group, we prepared the DEP medium following the same recipe, with the addition of 1% glucose. The glucose-supplemented group was exposed to glucose for 40 min prior to the DEP experiment. Figure 3 illustrates the cell culture and DEP experimental procedure.

**Figure 3:**
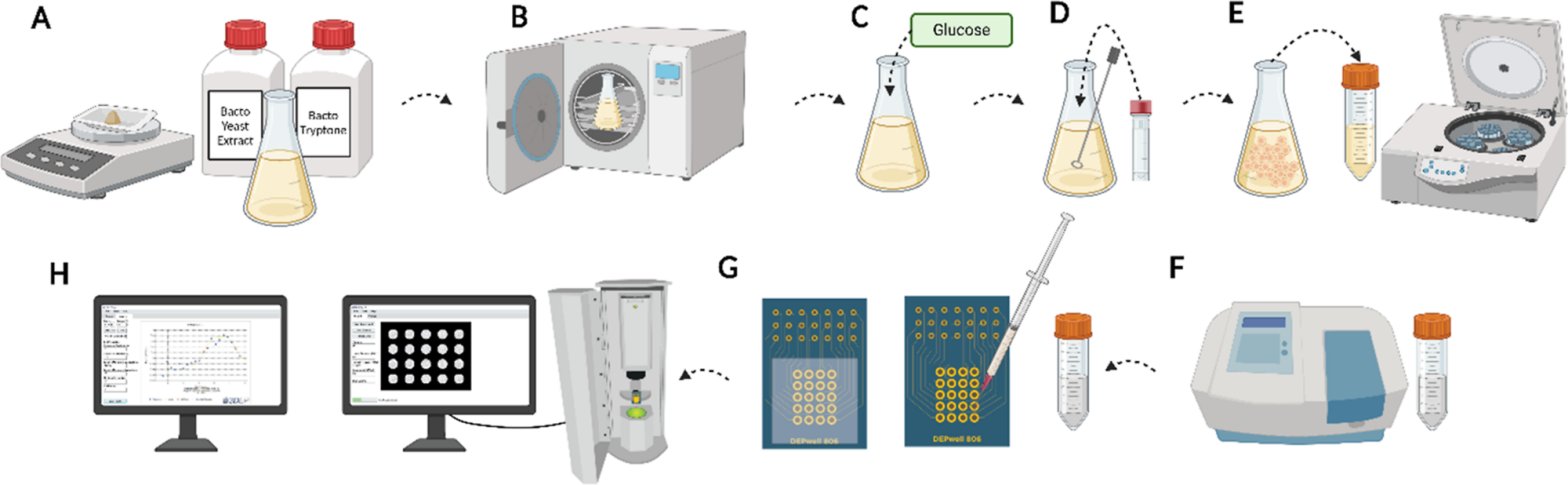
Cell culture and DEP experiment process. (A) Preparing UFTYE media, (B) autoclaving the media, (C) adding 1% (wt/vol) glucose, (D) Inoculating the media with CA1100, (E) washing the cells with NaCl solution and resuspending the cells in DEP buffer, (F) adjusting the cell optical density to 0.6–0.8 using spectrophotometer, (G) loading the DEP chip using 1 mL syringe and 25G needle, (H) running DEP experiment using 3DEP analyzer. Figure created with Biorender.com.

Before initiating the DEP experiment, the cells were washed once with 5 mL of DEP medium and subsequently resuspended in 5 mL of the same medium. Approximately 80 μL of the cell suspension was injected into the DEP chip (DEPwell 806, DEPtech, Uckfield, UK) using a 1 mL syringe fitted with a 25G needle (Air-Tite, USA). A 25 × 25 mm glass coverslip was placed over the chip to prevent meniscus formation. The chip was then inserted into the 3DEP system (DEPtech, Uckfield, UK), ensuring the door was properly closed to minimize exposure to ambient light. The light intensity on the 3DEP system was adjusted to 24. The frequency range and voltage were set to 0.5–45,000 kHz and 20 V_pp_, respectively. The experiment was run for 60 s, with an analysis duration of 30 s. In the 3DEP equipment software, the medium permittivity was set to 78 F/m, the conductivity to 0.008 S/m, and the cell radius was specified as 0.52 μm for the glucose-limited group and 0.8 μm for the glucose-supplemented group, based on SEM measurements.

### 3.5 Data Collection and Statistical Analysis

Experimental data for the glucose-limited group were collected first, followed by data collection for the glucose-supplemented group. Data from 10 technical replicates for each group were gathered using the 3DEP analyzer, with an *R*-value exceeding 0.9.

To obtain the real part of the Clausius–Mossotti plot, we utilized a MATLAB code (Supporting Information, S3) based on the single-shell dielectric model. The input parameters included the mean dielectric properties of both the glucose-limited and glucose-supplemented groups, as well as cell radius and shell thickness, which were measured using scanning and transmission electron microscopies. By applying the single-shell model in eq 4 discussed previously, the MATLAB code generated the real part of the Clausius–Mossotti plot for both groups.

GraphPad Prism 10.4.1 (GraphPad Software, MA) was used to conduct the *t*-test and one-way ANOVA to establish the significance of a glucose-supplemented versus a glucose-limited environment. Differences with *p* < 0.05 were considered statistically significant. The following notations *, **, ***, and **** describe the statistical difference with *p* values corresponding to *p* < 0.05, *p* < 0.01, *p* < 0.001, and *p* < 0.0001, respectively. The data were then plotted, with the standard error of the mean (SEM) displayed.

## 4 Results and Discussion

### 4.1 Kinetic Growth Study

To evaluate the effect of glucose on *C. auris* (CA1100) growth dynamics, growth kinetic analysis was conducted under two conditions: UFTYE medium without glucose and UFTYE medium supplemented with 1% (w/v) glucose. The growth curves (Supporting Information, Figure S1) for both conditions were determined by measuring the optical density at 600 nm (OD_600_) every 10 min for 48 h. The following kinetic parameters were calculated: lag time, maximum growth rate (μ_max_), and growth potential (area under the curve—AUC). As shown in Figure 4, the addition of merely 1% (w/v) glucose highly influenced the growth parameters of *C. auris* with a high statistical significance of *p* < 0.0001.

**Figure 4:**
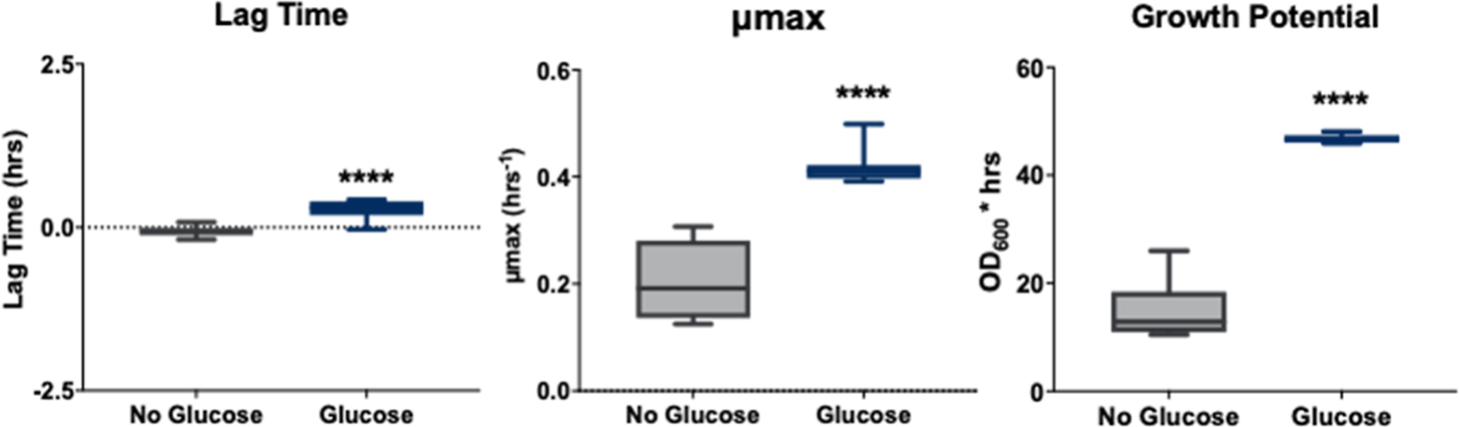
Kinetic growth analysis of lag time, μ_max_, and growth potential of *C. auris* (CA1100) in a glucose-limited and glucose-supplemented environment, indicating *p* < 0.0001.

The lag time represents the initial growth phase. In this phase, the cells adapt to their surrounding environment before the exponential growth phase begins.^13,37,38^ In Figure 4, it was observed that the lag time for *C. auris* cultured without glucose was notably shorter in comparison to the glucose-supplemented condition. Consequently, the cells in the glucose-limited medium displayed a more rapid adaptation and commenced division earlier than those in the glucose-supplemented medium. Furthermore, the longer lag time in the presence of glucose may indicate a delay in metabolic adjustments due to glucose uptake and utilization by the cells.

The maximum growth rate (μ_max_) is the fastest rate at which the cell population increases. On the growth curve, μ_max_ corresponds to the steepest slope observed during the exponential growth phase.^38,39^ Results show that despite the longer lag time, the *C. auris* cultured with 1% glucose-supplement exhibited a higher μ_max_, (0.42 ± 0.03 h^–1^) than the glucose-limited group (0.20 ± 0.07 h^–1^). This higher μ_max_ suggests that while adding glucose delays the onset of growth, it ultimately accelerates the growth rate during the exponential phase. This can be due to more efficient energy production and nutrient utilization.

Growth potential, measured as the area under the curve (AUC), quantifies the overall biomass accumulation over the 48 h incubation period. It is expressed in optical density units multiplied by time in hours (OD_600_·h) and calculated by integrating the area under the growth curve across the entire time course. Figure 4 illustrates the *C. auris* growth potential when grown under two conditions: glucose-supplemented and glucose-limited. This graph demonstrates that the cell’s overall growth in the glucose-supplemented group was much more significant than that of the control (glucose-limited). The AUC increased from 15 ± 5 OD_600_·h in the absence of glucose to 47 ± 1 OD_600_·h with glucose. This significant increase in growth potential indicates that glucose-supplemented *C. auris* cells grow by increasing their metabolic activity. Results of *C. auris* growth parameters demonstrate that even though adding glucose extended the lag time, it enhanced the maximum growth rate and overall growth potential, leading to a higher cell accumulation after the 48 h kinetic growth study.

### 4.2 Electron Microscopy

Both scanning and transmission electron microscopy were employed to measure the radius, cell wall, and membrane thickness of *C. auris* (CA1100) cells exposed to glucose-limited and glucose-supplemented environments. These measurements were incorporated into the single-shell model (Figure 2) to calculate the dielectric signatures (Figure 6A).

**Figure 5:**
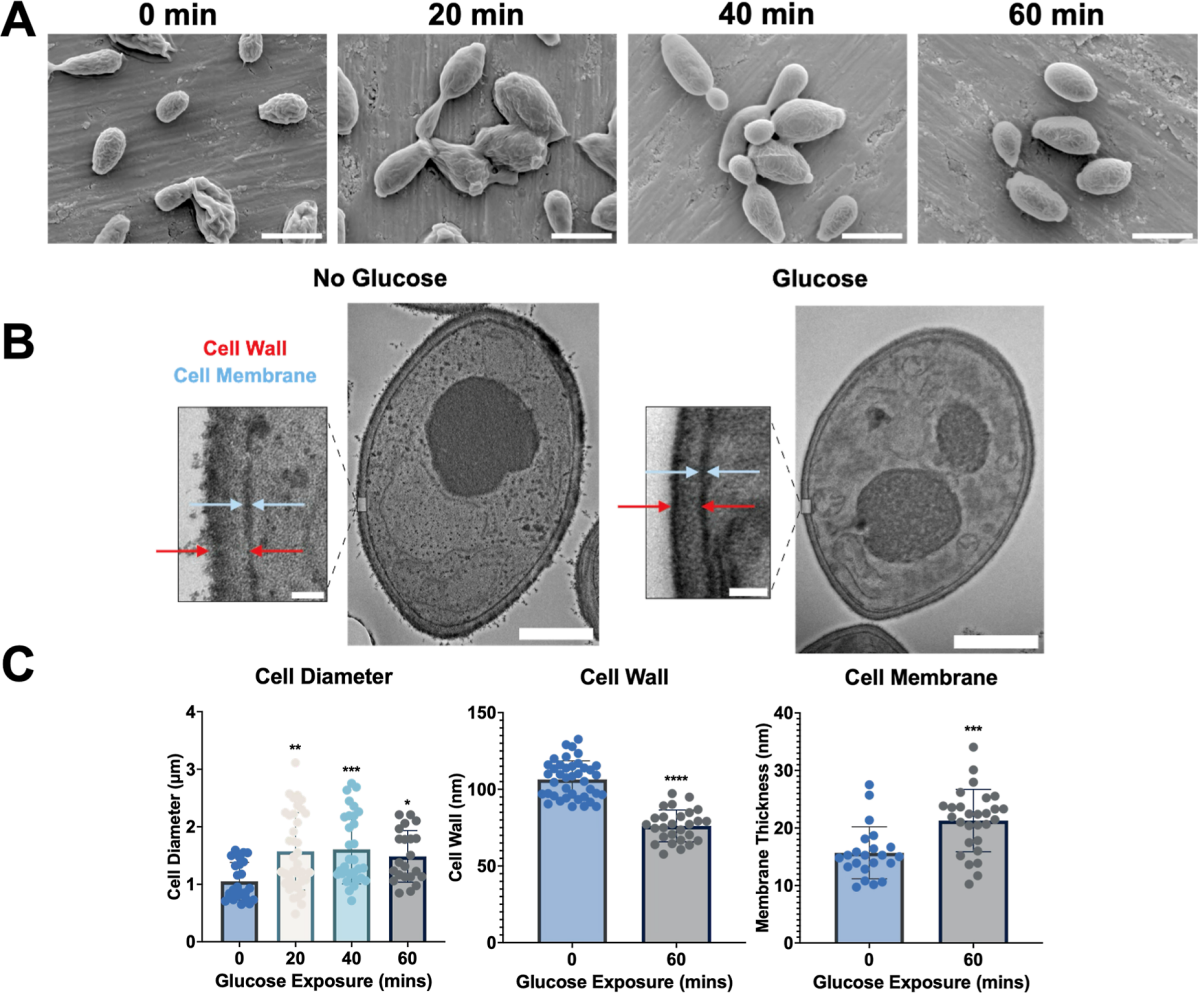
Morphological and intracellular study of *C. auris* (CA1100) cells in glucose-limited and glucose-supplemented environments. (A) SEM images at 0, 20, 40, and 60 min intervals after exposure to 1% (wt/vol) glucose. (SB: 3 μm) (B) representative single-cell TEM images of *C. auris* without prior exposure to glucose and after 60 min exposure to 1% (wt/vol) glucose. (SB: larger image, 1 μm; cropped image, 100 nm) (C) data for the cell diameter, cell wall, and membrane thickness of *C. auris* before and after glucose exposure.

**Figure 6:**
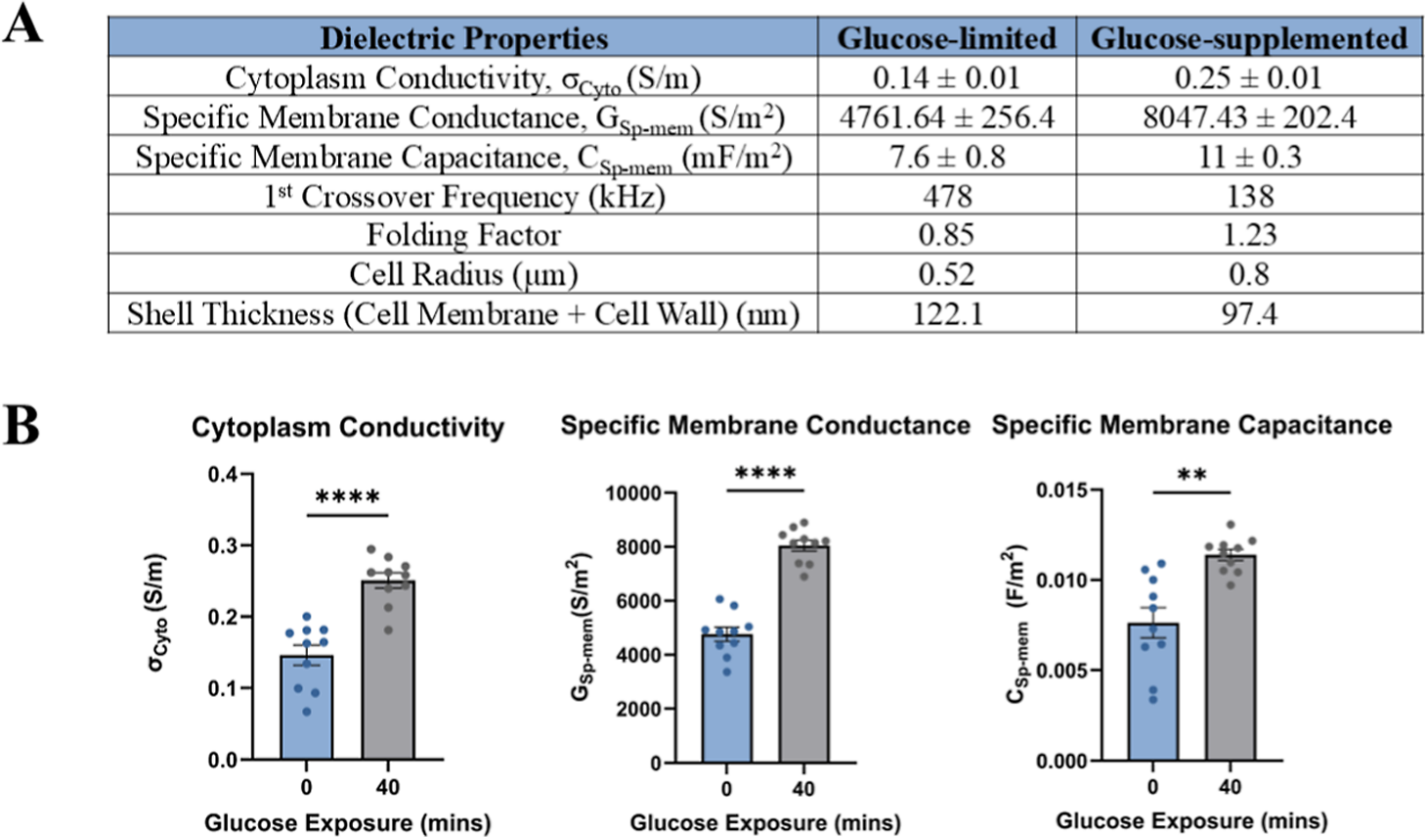
(A) Data summary of glucose-limited and glucose-supplemented groups using the single-shell model at 0.008 S/m medium conductivity, (B) statistical analysis of cytoplasm conductivity (*p* < 0.0001), specific membrane conductance (*p* < 0.0001) specific membrane capacitance (*p* = 0.0021).

First, SEM was performed to determine the size of *C. auris* cells. SEM images of *C. auris* (CA1100) cells were captured after different glucose exposure periods (0, 20, 40, and 60 min) (Figure 5A, Supporting Information, Figure S2). Using ImageJ software, multiple measurements of cell diameters were taken to calculate an average for each exposure time. These results were graphed and statistically analyzed using GraphPad Prism 10.4.1 software (Figure 5C) to compare the cell diameter across different exposure conditions.

The cells displayed the smallest average diameter at 0 min, i.e., before glucose exposure. After 20 and 40 min of glucose exposure, the cells showed a significant increase in diameter, with these exposure times exhibiting the largest average sizes among the samples. After 60 min, the cell diameter slightly decreased compared to the 20 and 40 min measurements, although it remained larger than the initial sample without glucose exposure, i.e., 0 min. These results indicate how *C. auris* cell morphology changes based on glucose availability.

Second, TEM samples were prepared and fixed at two distinct time points: without prior exposure to glucose, corresponding to the 0 min glucose condition, and after 60 min of glucose exposure. These fixed samples were then imaged, and multiple measurements of the cell wall and cell membrane (Figure 5B) were taken using ImageJ software. The data were graphed (Figure 5C) and analyzed for statistical significance to compare the internal properties of glucose-limited (0 min) and glucose-supplemented (60 min) samples. The quantitative analysis was derived from at least three independent cells per condition, providing a more representative measure of average thickness.

The results showed that the glucose-limited sample had a larger average cell wall thickness than the glucose-supplemented sample. However, the membrane thickness had larger measurements in the glucose-supplemented sample after 60 min of glucose exposure. These structural differences likely reflect the cell’s physiological adaptation to nutrient availability. Under stressful conditions, such as glucose limitation, *C. auris* cells have been reported to conserve resources and reinforce their wall as a protective response. This process involves significant remodeling and rebuilding of the outer layer, which can lead to a thickened cell wall.^39,40^ Because the cell wall and membrane were treated as a single shell in our dielectric model, these structural differences are expected to influence the electrical behavior of the cells. Specifically, changes in wall thickness and membrane composition can alter the effective shell parameters, thereby affecting how cells interact with external electric fields. This relationship is further explored in the dielectric characterization section.

### 4.3 Dielectric Characterization

This section presents the results of the dielectric signatures obtained for the two *C. auris* (CA1100) cell groups: glucose-limited and glucose-supplemented conditions after 40 min of glucose exposure at 80 μS/cm medium conductivity conditions. The cell dielectric properties were curve-fitted using a single-shell dielectric model. The single-shell dielectric model assumes a spherical cell geometry and a homogeneous cytoplasmic region enclosed by a membrane with distinct dielectric properties. We used this model because *C. auris* cells are generally ellipsoidal to near spherical shape. The assumption of a homogeneous internal dielectric environment is reasonable given that the dielectric measurements are dominated by bulk cytoplasmic behavior rather than suborganelle variability. Additionally, the single-shell model provides a balance between model simplicity and fitting accuracy, as supported by prior work modeling yeast and fungal cells.^41,42^ The summary of the results is shown in Figure 6A.

The dielectric properties of *C. auris* (CA1100) cells were assessed after 40 min of glucose exposure. The cytoplasm conductivity, specific membrane conductance, and specific membrane capacitance were compared and statistically analyzed between the glucose-limited and glucose-supplemented groups.

Results demonstrated a statistically significant increase (*p* < 0.0001) in cytoplasm conductivity for the glucose-supplemented group compared to the glucose-limited group. The mean cytoplasm conductivity for the glucose-limited group was 0.14 S/m, while the glucose-supplemented group showed a mean of 0.25 S/m. The glucose-supplemented group demonstrated a statistically significant increase (*p* < 0.0001) in specific membrane conductance compared to the glucose-limited group. A significant increase was seen in the mean conductance of the glucose-supplemented group, 8047.4 F/m^2^ (*p* < 0.0001), when compared to the glucose-limited group, with a mean conductance of 4761.6 F/m^2^. The specific membrane capacitance was also significantly higher in the glucose-supplemented group compared to the glucose-limited group (*p* = 0.0021). Figure 6B represents the statistical analysis of dielectric signatures of control (glucose-limited) and positive (glucose-supplemented) groups.

These results indicate that glucose exposure significantly alters the dielectric signatures of *C. auris*, with significant increases in cytoplasm and membrane electrical properties in the glucose-supplemented group. The observed increase in maximum specific growth rate, lag time, and growth potential in glucose-supplemented conditions directly correlates with changes in the dielectric properties of *C. auris*. The higher μ_max_ indicates an accelerated metabolic rate, leading to increased ion transport and enzymatic activity, enhancing cytoplasmic conductivity. This heightened metabolic state results in a greater accumulation of intracellular ions, contributing significantly to the changes in the overall dielectric response.

The extended lag phase suggests that *C. auris* undergoes metabolic adjustments before entering exponential growth. During this phase, the cells are actively modifying their membrane composition and intracellular ion concentrations to adapt to the glucose-rich environment. These biochemical changes alter membrane properties and cytoplasmic conductivity, affecting the cell’s interaction with external electric fields. Furthermore, the increase in growth potential signifies enhanced cell viability and division, leading to higher cell density and collective ionic activity within the population. This increase in cellular interactions and extracellular ion exchange further impacts dielectric dispersion patterns.

Additionally, the frequency-dependent dielectric response of *C. auris* was evaluated for both the glucose-limited and glucose-supplemented groups. The real part of the Clausius–Mossotti factor, *Re*[*K*(ω)], calculated using eq 2, was plotted as a function of AC frequency (Hz) at a fixed peak-to-peak voltage of 20 V_pp_ and 0.008 S/m medium conductivity for both groups using MATLAB.

The first crossover frequency, where the real part of the Clausius–Mossotti factor, *Re*[*K*(ω)], transitions from negative to positive dielectrophoresis (negative to positive axis shift), was observed for both the glucose-limited and glucose-supplemented groups. In the glucose-limited group, the first crossover occurred at ∼478 kHz (Black curve). In contrast, the glucose-supplemented group exhibited a lower crossover frequency at ∼138 kHz (blue curve) as shown in Figure 7.

**Figure 7:**
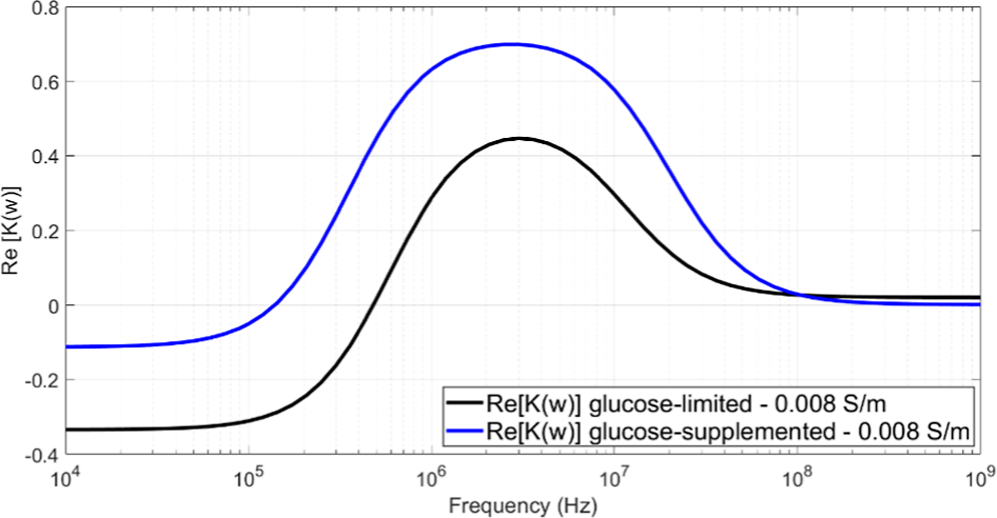
Real part of the Clausius–Mossotti factor plot for glucose-limited and glucose-supplemented groups at 0.008 S/m medium conductivity and a fixed peak-to-peak voltage of 20 V_pp_. The glucose-limited and glucose-supplemented groups demonstrated the 1st crossover frequency at 478 kHz and 138 kHz, respectively.

This shift in the first crossover frequency suggests that the cells in the glucose-supplemented group, after glucose exposure, begin to demonstrate positive dielectric behavior at a lower frequency compared to the glucose-limited group. The change in the first crossover frequency is mainly pertinent to the membrane morphology and characteristics, suggesting that the effect of glucose is a surface phenomenon and does not affect the intracellular contents significantly. The increase in cellular interactions and extracellular ion exchange shifts the crossover frequency following glucose treatment.

## 5 Conclusions and Future Work

In this study, we cultivated *C. auris* strain CA1100 to investigate its growth kinetics and dielectric signatures before and after glucose exposure, recognizing glucose as a key energy source for *C. auris*. The lag time, maximum growth rate, and growth potential all demonstrated a significant increase following glucose exposure. The results presented above demonstrate that exposure to glucose significantly modifies the dielectric properties of *C. auris* (CA1100) cells. The glucose-supplemented group showed higher cytoplasm conductivity, specific membrane conductance, and specific membrane capacitance compared to the cells in the glucose-limited group, indicating elevated cellular polarization following glucose treatment.

Moreover, the plot of the real part of the Clausius–Mossotti factor demonstrated that the glucose-supplemented group exhibited a lower first crossover frequency, suggesting that glucose exposure alters cellular electrical properties, resulting in increased dielectric activity at lower frequencies. These findings underscore the influence of glucose on the membrane and cytoplasmic properties of *C. auris*.

While the current findings establish feasibility in *C. auris*, we acknowledge that diagnostic specificity cannot be claimed until direct comparisons are made with closely related *Candida* species (e.g., *Candida albicans*, *Candida glabrata*) and bacterial pathogens. As such, our next phase of work will focus on comparative DEP signature mapping across these organisms under identical conditions to validate the uniqueness, specificity, and clinical utility of the *C. auris* dielectric fingerprint.

Additionally, for future studies, we aim to investigate the effects of various antifungal agents on the dielectric properties of *C. auris*. By exposing CA1100 to different antifungals, we will analyze how these treatments influence dielectric properties. This work will provide insights into the mechanisms by which antifungal agents modify the electrical properties of *C. auris*, leading to the development of new therapeutic strategies to combat fungal infections more effectively.

## Supplementary Material



## Data Availability

The data that support the findings of this study are available from the corresponding author upon request.

## Notes

Additional data and figures supporting this study are available in the Supporting Information. These include 48 h growth curves for glucose-limited and glucose-supplemented conditions, SEM images at 0, 20, 40, and 60 min intervals after exposure to 1% (wt/vol) glucose, and MATLAB code was used to obtain the real part of the Clausius–Mossotti factor plot (PDF)
