# Supplementary material for: Dielectrophoretic Profiling of Candidozyma auris: the Effect of Glucose on Cellular Polarizability
Source: ACS Meas Sci Au. 2025 Sep 26;5(6):805–13. doi: 10.1021/acsmeasuresciau.5c00084 (PMC12715728; doi:10.1021/acsmeasuresciau.5c00084)
Supplement: Supplementary file 1 [file tg5c00084_si_001.pdf]

Supplemental Information

Dielectrophoretic Profiling of *Candidozyma auris*: The Effect of Glucose on Cellular Polarizability

Negar Farhang-Doost[a], Camila S. Cué Royo[b], Tagbo H. R. Niepa[b, c], Soumya K. Srivastava[a]*

[a]Department of Chemical and Biomedical Engineering, West Virginia University, Morgantown, WV 26505, United States.

[b]Department of Chemical Engineering, Carnegie Mellon University, Pittsburgh, PA 15213, United States.

[c]Department of Biomedical Engineering, Carnegie Mellon University, Pittsburgh, PA 15213, United States.

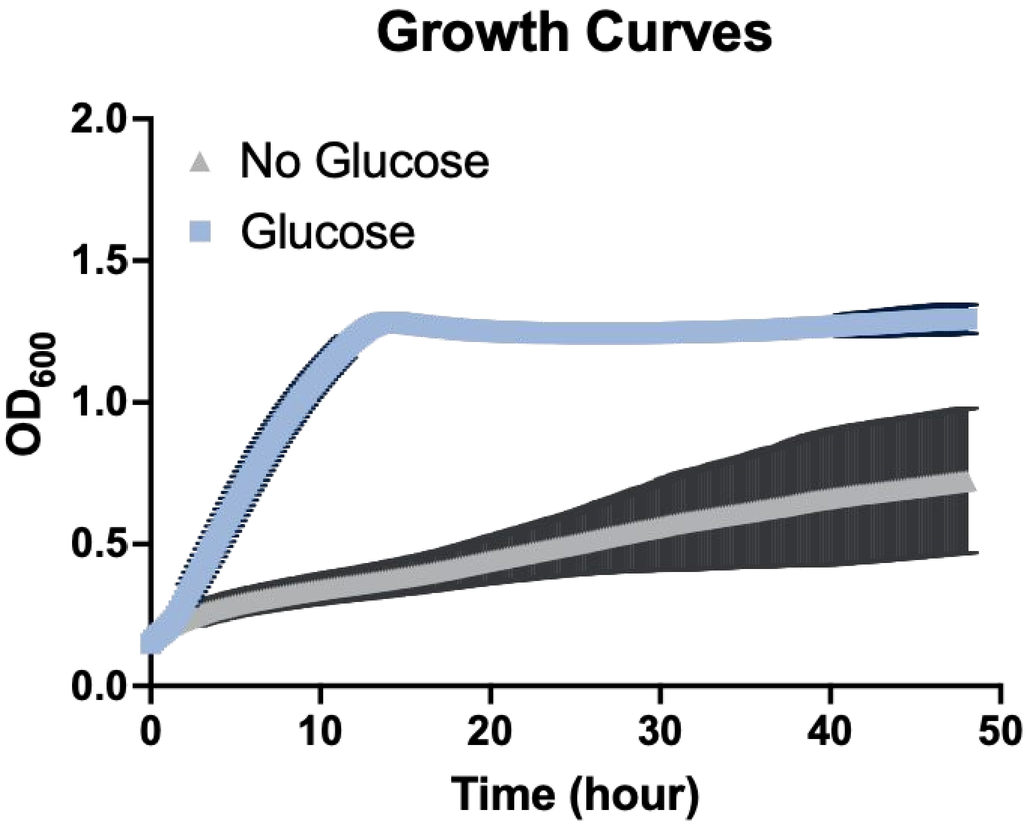

Figure S1 *Candidozyma auris CA1100 48-hour growth curves for two conditions (glucose-limited and glucose-supplemented)*

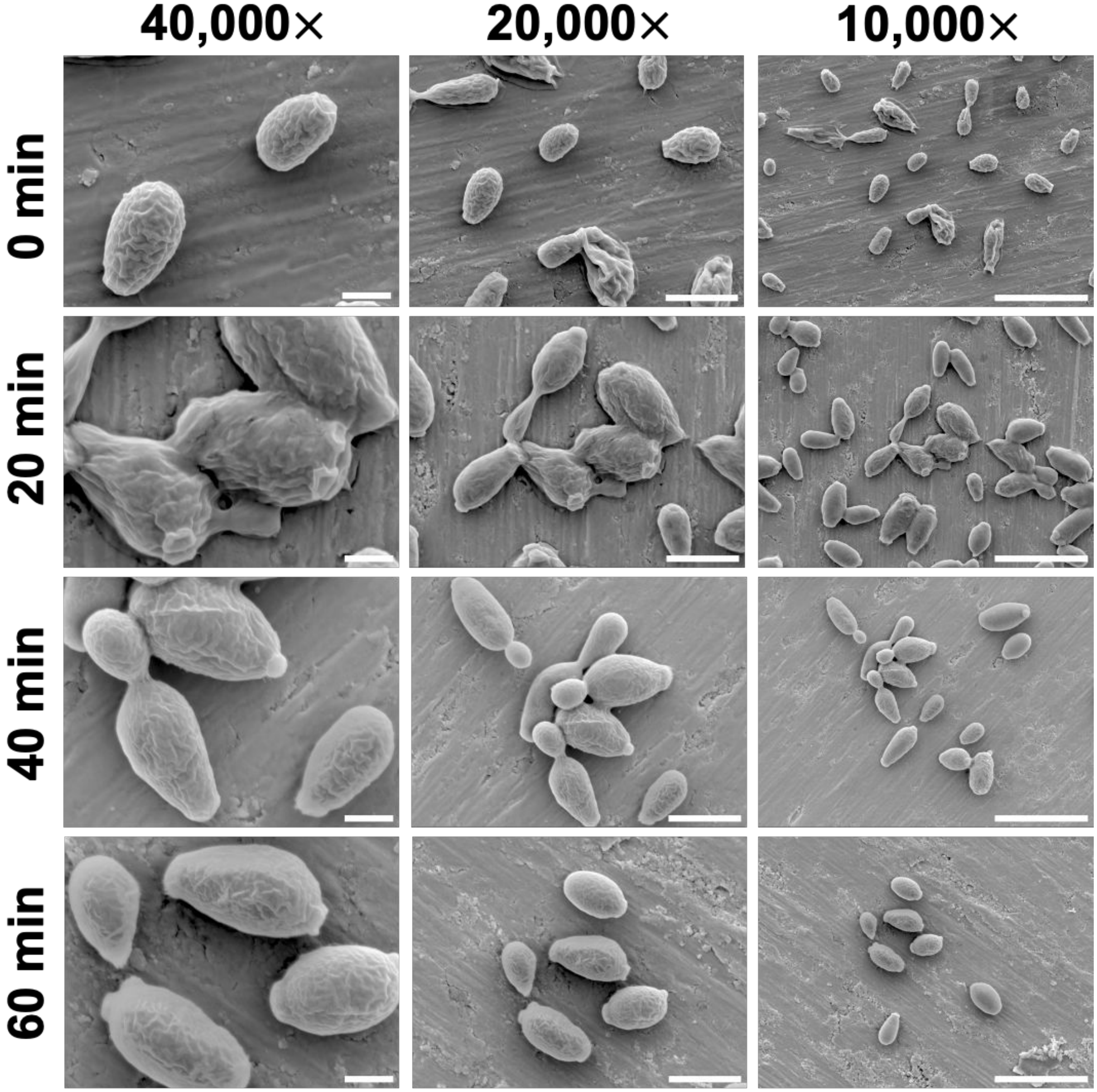

Figure S2 *Candidozyma auris CA1100 SEM images at 0, 20, 40, and 60 minutes intervals after exposure to 1% (wt/vl) glucose. (SB: 1 µm for 40,000x, 3 µm for 20,000x, and 5 µm for 10,000x magnification, respectively.)*

S3: MATLAB Code

```
d2=1.051e-6;
r2=d2/2;
d=0.1221e-6;
r1=r2-d;
a=(r2/r1)^3;
d2i=1.6e-6;
r2i=d2i/2;
di=0.0974e-6;
r1i=r2i-di;
ai=(r2i/r1i)^3;
e0=8.854e-12;
j=sqrt(-1);
f1=[1 1.2 1.44 1.2^3 1.2^4 1.2^5 1.2^6 1.2^7 1.2^8 1.2^9 1.2^10 1.2^11 1.2^12];
w=2*pi*[1e3*f1 1e4*f1 1e5*f1 1e6*f1 1e7*f1 1e8*f1 1e9*f1];
f0=w ./(2*pi);
q31=0.008;
e3=78*e0;
e1=60*e0;
q11=0.1460004779;
q11i=0.2508215744;
G21=4761.647608;
q21=G21*d;
G21i=8047.439099;
q21i=G21i*di;
C21=0.007631264191;
e21=C21*d;
C21i=0.01138832903;
e21i=C21i*di;
tau = 0;
scale=0 ;
factor=1 ;
ec11=e1-j*q11 ./w;
ec21=e21-j*q21 ./w;
ec31=e3-j*q31 ./w;
ec11i = e1-j*q11i./w;
ec21i = e21i-j*q21i./w;
ec31i = e3-j*q31./w;
dispersion=scale*(1./(1+j.*tau.*w));
ec121=(ec11-ec21) ./(ec11+2*ec21);
ef21=ec21 .*(a+2*ec121) ./(a-ec121);
cmf1= (dispersion+ ((ef21-ec31) ./(ef21+2*ec31)));
re1= real(cmf1);
Geff1=q21/d;
Ceff1=e21/d;
ec121i=(ec11i-ec21i)./(ec11i+ (2*ec21i));
ef21i=ec21i .*(ai+2*ec121i)./(ai-ec121i);
cmf1i= (dispersion + ((ef21i-ec31i)./(ef21i +(2*ec31i))));
re1i= real(cmf1i);
Geff1i = q21i/di;
```

```
Ceff1i = e21i/di;
figure(1)
semilogx(f0,re1,'k', f0,re1i,'b','LineWidth',10);
xlim([10000 1000000000]);
xlabel('Frequency (Hz)','FontName', 'Arial','FontSize', 20);
ylabel('Re [K(w)]','FontName', 'Arial','FontSize', 20);
legend ({'Re[K(w)] no glucose - 0.008 S/m' 'Re[K(w)] glucose - 0.008 S/m'},'FontName',
'Arial','FontSize', 20,'LineWidth',1);
set(gca, 'FontSize', 16)
grid on
```
